# Identification of Differentially Expressed Proteins and Phosphorylated Proteins in Rice Seedlings in Response to Strigolactone Treatment

**DOI:** 10.1371/journal.pone.0093947

**Published:** 2014-04-03

**Authors:** Fangyu Chen, Liangrong Jiang, Jingsheng Zheng, Rongyu Huang, Houcong Wang, Zonglie Hong, Yumin Huang

**Affiliations:** 1 School of Life Sciences, Xiamen University, Xiamen, China; 2 Department of Plant, Soil, and Entomological Sciences, and Program of Microbiology, Molecular Biology and Biochemistry, University of Idaho, Idaho, United States of America; Nanjing Agricultural University, China

## Abstract

Strigolactones (SLs) are recently identified plant hormones that inhibit shoot branching and control various aspects of plant growth, development and interaction with parasites. Previous studies have shown that plant D10 protein is a carotenoid cleavage dioxygenase that functions in SL biosynthesis. In this work, we used an allelic SL-deficient *d10* mutant XJC of rice (*Oryza sativa* L. spp. *indica*) to investigate proteins that were responsive to SL treatment. When grown in darkness, *d10* mutant seedlings exhibited elongated mesocotyl that could be rescued by exogenous application of SLs. Soluble protein extracts were prepared from *d10* mutant seedlings grown in darkness in the presence of GR24, a synthetic SL analog. Soluble proteins were separated on two-dimensional gels and subjected to proteomic analysis. Proteins that were expressed differentially and phosphoproteins whose phosphorylation status changed in response to GR24 treatment were identified. Eight proteins were found to be induced or down-regulated by GR24, and a different set of 8 phosphoproteins were shown to change their phosphorylation intensities in the dark-grown *d10* seedlings in response to GR24 treatment. Analysis of these proteins revealed that they are important enzymes of the carbohydrate and amino acid metabolic pathways and key components of the cellular energy generation machinery. These proteins may represent potential targets of the SL signaling pathway. This study provides new insight into the complex and negative regulatory mechanism by which SLs control shoot branching and plant development.

## Introduction

Strigolactones, a group of carotenoid-derived terpenoid lactones, are recently identified endogenous plant hormones that inhibit shoot branching [1], [2]. Tillers in rice (*Oryza sativa* L.) are derived from vegetative shoot branching, and the growth of tillers is one of the most important agronomic traits for rice grain production. It has been shown that monocot and dicot species share a conserved pathway for the biosynthesis of SLs [3]–[6]. In the last decade, remarkable progress has been made in understanding the molecular basis of shoot branching through studies of a series of increased branching mutants in the SL pathway, including *more axillary growth* (*max*) of *Arabidopsis*, *ramosus* (*rms*) of pea (*Pisum sativum*), *decreased apical dominance* (*dad*) of petunia (*Petunia hybrida*), and *dwarf* (*d*) or *high-tillering dwarf* (*htd*) of rice [3]–[5], [7]–[20]. Three rice genes, *D10, D17/HTD1* and *D27*, have been implicated in the SL synthesis. D27 is an iron-binding protein with the *β*-carotene isomerase activity that converts *trans*-*β*-carotene into 9-*cis*-*β-*carotene. D17/HTD1 encodes a carotenoid cleavage dioxygenase 7 (OsCCD7) that cleaves 9-*cis*-*β-*carotene into a 9-*cis*-configured aldehyde. D10 encodes another type of carotenoid cleavege dioxygenase (OsCCD8b) that incorporates three oxygens into 9-*cis*-*β*-apo-10′-carotenal and performs molecular rearrangement, linking carotenoids with SLs and producing carlactone, a compound with SL-like biological activities [4], [21], [22]. In addition, two other proteins, DWARF3 (D3) and DWARF14 (D14) have been proposed to function in the perception of the SL signal. D3 is an F-box protein, while D14/D88/HTD2 is a member of the hydrolase superfamily [3]–[5], [15], [18]. Recently, protein structure analysis has revealed that D14 forms an α/β-fold that contains a canonical catalytic triad with a large internal cavity capable of binding SLs [23]–[25].

SLs have been demonstrated to be important endogenous regulators of plant growth and development both underground and aboveground [26]. Other notable functions of SLs include seed germination [27], [28], root growth and architecture [13], [29], [30], adventitious root formation [31], flower development [13], leaf senescence and development [8], [11], [13], [32]. Genes implicated in the SL biosynthesis and signaling pathways have been shown to express in mature seeds and young seedlings as well as adult plants, suggesting that they play various roles in a spectrum of physiological and developmental processes ranging from germination to leaf development [33].

The mesocotyl of young rice seedlings is a tissue located between the coleoptilar node and a basal part of the seminal root. Previous studies have suggested that the elongation of mesocotyl is controlled by multiple genetic, developmental and environmental signals [34]–[37]. Rice mutants defective in SL-related genes (*d3*, *d10*, *d14*, *d17* and *d27*) have been shown to produce longer mesocotyl than the wild type seedlings when grown in darkness [38]. Exogenous application of a synthetic SL analog, GR24, can restore the normal mesocotyl phenotype in the SL-deficient mutants (*d10*, *d17* and *d27*). Regardless of the remarkable progress that has been made, the molecular mechanisms by which SLs control shoot branching and other developmental and physiological processes still remain to be elucidated.

Two-dimensional gel electrophoresis (2-DE) is a powerful proteomics technique for differential display of proteins with posttranslational modifications. It can separate thousands of proteins based on their differences in charge and size [39], [40], and has been used to monitor molecular responses induced by the activation or inhibition of specific signaling pathways [41]. Quantitative proteomics analyses have identified a large number of brassinosteroid (BR)-regulated proteins, including the BR signaling components, which are regulated by phosphorylation at the posttranslational level [42]–[44]. Two of these BR signaling components, BSK1 and BSK2, have been identified through proteomic characterization of phosphorylated proteins of the BR-deficient mutant *det-2* in response to BR treatment [44]. The identification of these additional components has filled the last gap in the BR signal pathway [44].

Protein phosphorylation and phosphorelay have been recognized as an important mechanism for signal transduction. Among the several ways to detect phosphoproteins, the application of antibodies specific to phosphotyrosine, phosphothreonine, or phosphoserine is advantageous [45]. In this study, we found that mesocotyl elongation of dark-grown rice seedlings was higher in the *d10* mutant than in the wild-type plant, and this dark-hypersensitivity could be rescued by exogenous application of GR24. To understand the molecular mechanism underlying the function of SLs in dark-grown *d10* seedlings, we analyzed the differential expressed proteins and phosphoproteins in *d10* in response to SL treatment, and identified several candidates that may play a role in the SL responses in rice.

## Results

### Rescue of the tillering phenotype in XJC by GR24

**Figure 1:**
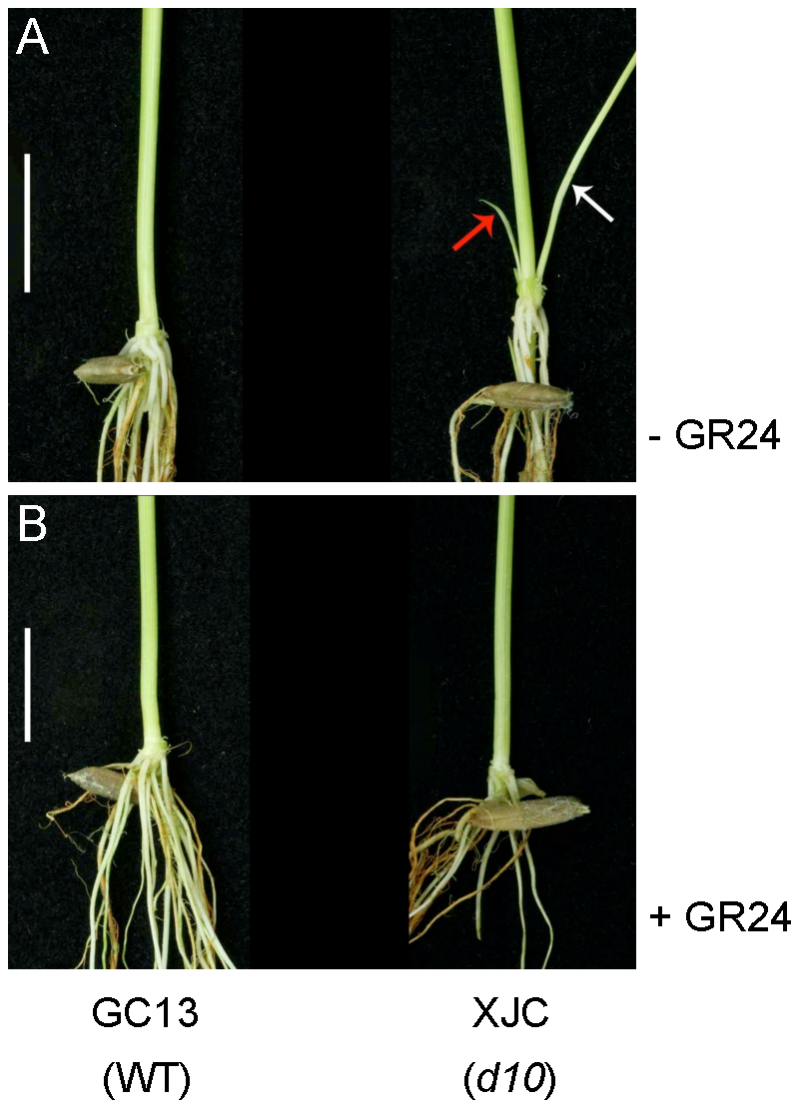
Response of *d10* mutant and wild type seedlings to the application of GR24. Wild type (WT) GC13 and mutant XJC (*d10*) seedlings were grown in the presence of 1.0 μM GR24 for two weeks. Tillers were present in the axil of the first (red arrow) and second (white arrow) leaves of the mutant (a), but absent in WT plants and the GR24-treated *d10* mutant (b). The tiller outgrowth phenotype in *d10* was suppressed by application of GR24. Scale bars  = 1 cm.

*D10* gene expression is controlled by feedback regulation [4], indicating that the level of *D10* mRNA accumulation might be a critical step in the regulation of SL biosynthesis. Our previous study revealed that a 39 bp deletion at the second exon of *D10* (LOC_Os01g0746400, OsCCD8b) results in the frame-shift mutation in the *indica* rice mutant XJC, exhibiting a high-tillering dwarf phenotype [46]. Quantitative real-time reverse transcription-PCR (qRT-PCR) analysis also confirms the dramatic up-regulation of *D10* expression in XJC compared to that in the wild type GC13 [46]. By applying 1.0 μM GR24, a synthetic strigolactone analog, to the wild-type (GC13) and *d10* (XJC) seedlings in a hydroponic culture, we found that the exogenous supplement of GR24 was able to fully inhibit tiller bud outgrowth of 2-week-old *d10* seedlings (Figure 1), suggesting that the tillering phenotypes of *d10* mutant plants could be rescued by GR24 treatment. These findings further confirm that XJC is indeed defective in SL biosynthesis, while the perception and signaling pathway of SLs in XJC is apparently normal.

### Rescue of the mesocotyl elongation phenotype in XJC by GR24

**Figure 2:**
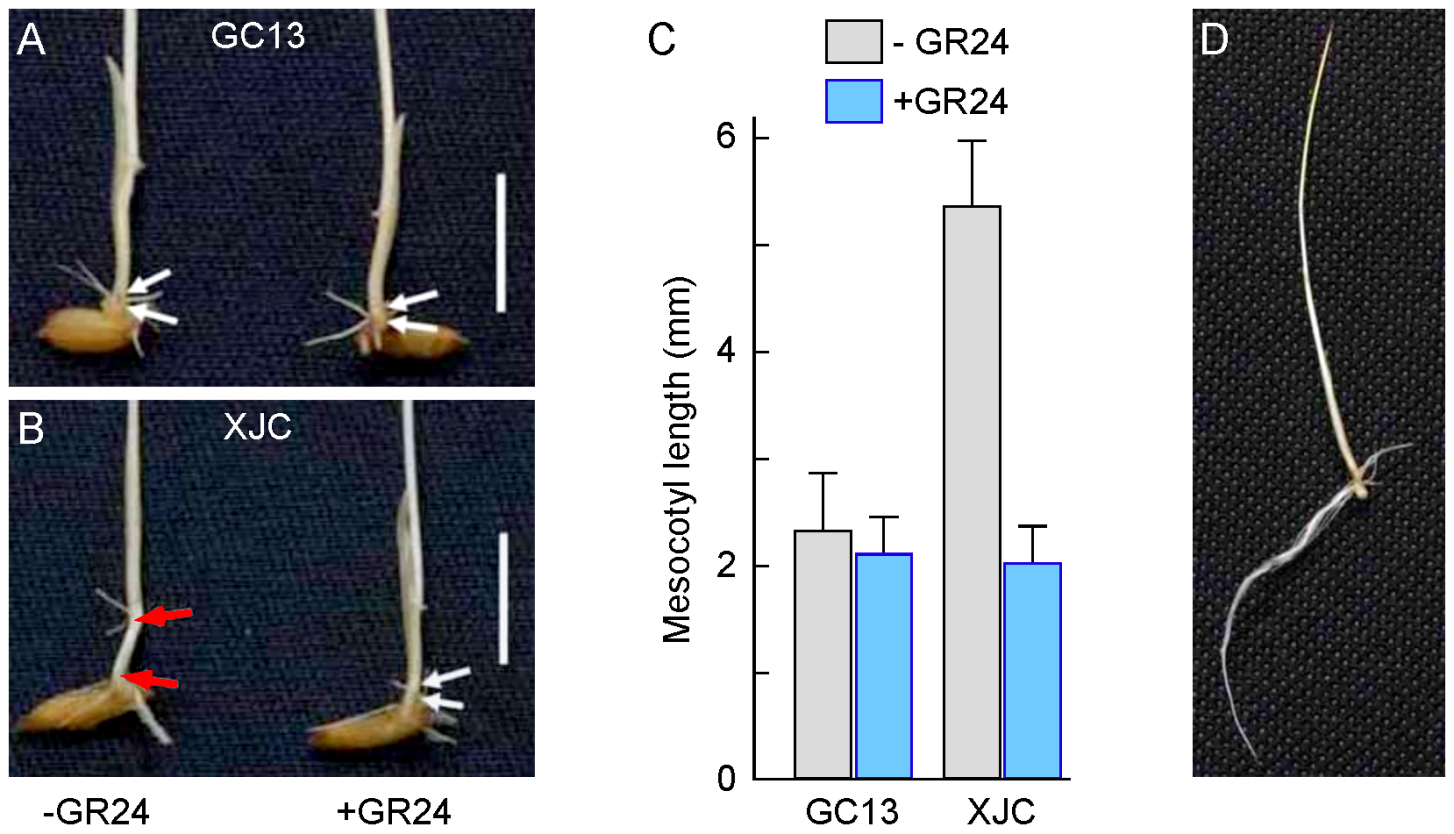
Effect of GR24 on mesocotyl elongation of *d10* mutant XJC seedlings. (a–b) Phenotypes of mesocotyl elongation in GC13 (wild type control) and XJC (*d10* mutant) seedlings grown in the absence (-GR24) or presence (+GR24) of 1.0 μM GR24 in darkness for 6 days. White arrows indicate the positions between the coleoptilar node and a basal part of the seminal root. Red arrows indicate the elongated mesocotyl. Bars  = 1 cm. (c) The length of mesocotyls of the wild type (GC13) and *d10* mutant (XJC) seedlings grown in the absence (-GR24) or presence (+GR24) of 1.0 μM GR24 in darkness for 6 days. Data are the means ± S.E. obtained from 12 seedlings. The ratio of mesocotyle length between the control (-GR24) and treatment (+GR24) with a *p*-value of less than 0.05 was considered to be statistically significant. The *p*-values of GC13 and XJC were 0.152995 and 1.15763E-07, respectively. (d) A representative etiolated seedling used for total protein extraction. The seedlings of *d10* mutant (XJC) seedlings were grown in presence (+GR24) of 1.0 μM GR24 in darkness for 6 days for total protein extraction.

To examine the effects of SLs in mesocotyl elongation, seedlings were germinated and grown on agar plates containing 0 and 1.0 μM of GR24 for 6 days in darkness. The length of mesocotyl of *d10* mutant XJC was 2.3 fold longer than that of the wild-type GC13 seedlings (Figure 2A–C). GR24 did not affect mesocotyl elongation of wild-type seedlings but decreased the length of mesocotyl of *d10* mutant XJC. At a concentration of 1.0 μM GR24, the length of mesocotyl between XJC and GC13 was indistinguishable (Figure 2A–C). This result suggests that mesocotyl length is negatively regulated by SLs in *d10* mutant seedling under dark-growth conditions.

### Identification of SL-responsive proteins

**Figure 3:**
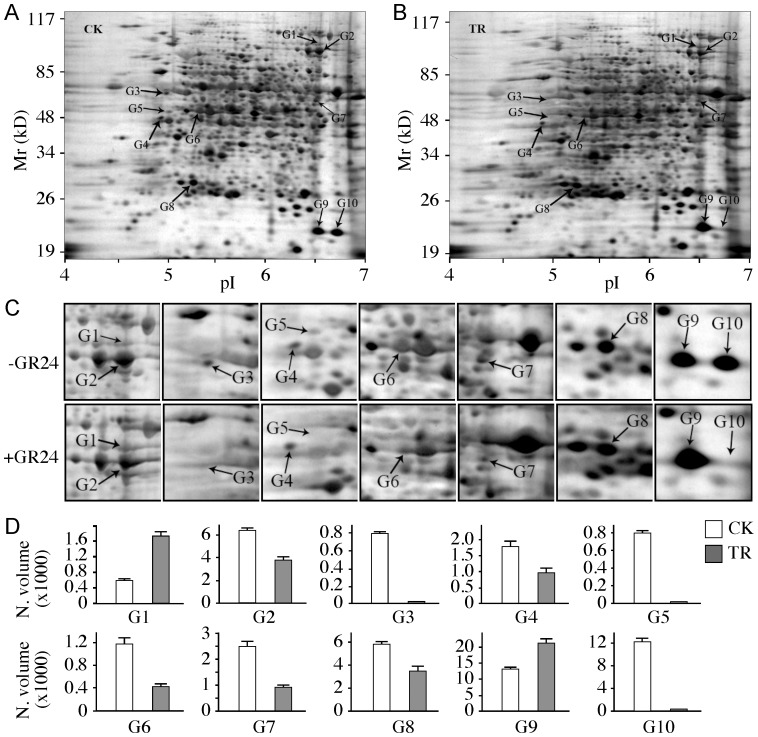
Differential expression of proteins in etiolated *d10* seedlings treated with GR24. (a–b) 2-DE maps of total proteins extracted from XJC seedlings grown in the absence of GR24 (CK, control) or 1.0 μM GR24 (TR). (c) Magnified images containing differentially expressed protein spots from G1 to G10. (d) Abundance analysis of the differentially expressed protein spots determined using the PDQuest 2D analysis software (BioRad). Normalized quantitative volumes of protein spots were exported from PDQuest and graphical presentations of protein spots were generated using Excel software. Normalized volumes (N. volume x1000) of protein spots are shown in *y*-axes. The empty histograms represent spot volume means of the control (CK) and the solid histograms represent the treatments (TR).

To examine the molecular mechanism of inhibition of GR24 on mesocotyl elongation in dark-grown *d10* mutant seedlings, we applied proteomic approach to the analysis of differentially expressed proteins and phosphoproteins in the *d10* mutant seedlings in response to GR24 application. Etiolated XJC seedlings after removal of residual seeds (Figure 2D) with or without GR24 treatment were harvested and their proteomes were resolved by 2-DE. Protein profiles of the gels were visualized with silver staining (Figure 3A–B). A total of 957±3(n = 3)protein spots were detected on each of the 2-DE maps. Ten proteins showed more than 1.5-fold reproducible changes in abundance (Figure 3C). After GR24 treatment in the XJC plants, two proteins (G1 and G9) were up-regulated, five (G2, G4, G6, G7 and G8) down-regulated, and three (G3, G5 and G10) became undetectable (Figure 3C–D). By means of mass spectrometry, seven protein spots (G1, G2, G3, G4, G6, G8 and G10) were successfully identified as eight different proteins (Table 1). Spot G4 was identified as the mixture of putative late embryogenesis abundant (LEA) protein and putative adenosine kinase (AK). Sucrose synthase 2 (SUS2) of spot G1 was up-regulated by GR24 treatment. The protein levels of putative vitamin B12-independent methionine synthase (MetE), LEA, AK, actin-7 (ACT7) and *L*-ascorbate peroxidase 2 (APX2) were down-regulated in XJC seedlings by exogenous SL application. Two protein spots, 26S proteasome regulatory subunit A (OsI_09330, G3) and mitochondrial phosphate translocator (MPT, G10) disappeared in XJC seedlings treated with GR24.

**Table 1:** Identification of differentially expressed proteins in response to SL treatment a.

Spot	GenBank Accession	Protein name (abbreviation)	Mr(Da)/pI^b^	MP^c^	SC (%)^d^	Mowse score^e^	Exp. score (*P*-value)^f^	Protein change^g^	FC±SE^h^	*p*-value^i^
G1	S19139	Sucrose synthase 2 (SUS2)	93286/5.9	23	25	83	0.00035	Up	2.9±0.1	0.0013
G2	Q2QLY4	Vitamin B12-independent methionine synthase (MetE)	84955/5.9	34	44	214	2.6E-17	Down	1.7±0.1	0.0027
G3	OsI_09330	26S proteasome regulatory subunit 6A	47044/4.9	16	36	71	0.0097	DA		
G4 (Mix)	Q75LD9	(1) Late embryogenesis abundant protein (LEA)	34777/4.9	19	59	133	3.3E-09	Down	1.8±0.1	0.0127
	Q6K1R5	(2) Adenosine kinase (AK)	37369/5.1	11	47	74	0.0029			
G5		No identification						DA		
G6	P0C542	Actin 7 (ACT7)	41873/5.2	23	67	167	2.7E-12	Down	2.9±0.1	0.0037
G7		No identification						Down	2.7±0.2	0.0062
G8	NP_001060741	*L*-Ascorbate peroxidase 2 (APX2)	27215/5.2	15	57	111	1.1E-06	Down	1.7±0.09	0.0080
G9		No identification						Up	1.7±0.06	0.0072
G10	Q9FMU6	Mitochondrial phosphate translocator (MPT)	40463/9.3	13	32	75	0.0015	DA		

a Rice *d10* mutant XJC seedlings were germinated and grown on medium in the presence of 1.0 μM GR24 for 6 days in darkness. Seedlings grown without exogenous application of GR24 served as a control. Total proteins were extracted from seedlings and resolved by 2-DE. Protein spots that exhibited differences in intensity were excised for identification by mass spectrometry.

b Relative molecular mass (Mr) in Da and isoelectric point (pI) of proteins are theoretical.

c Number of matched peptides (MP).

d Sequence coverage (SC, %) of the identified peptides.

e Proteins with a Mowse score of more than 61 were considered to be identified at a statistically significant level. Significance threshold *p*<0.05.

f Expectation scores are the probability-based values obtained from database search by MASCOT. Values less than their threshold (*p*<0.05) are statistically confident with more than 95% certainty.

g Protein spot changes after GR24 treatment was indicated by up-regulation (Up), down-regulation (Down) and disappearance (DA).

h Fold changes (FC) with standard errors (±SE) from three biological replicates were calculated as the ratio of normalized spot intensities between the treatment and control.

i Fold changes (FC) in protein spot intensity with a *p*-value of less than 0.05 were considered to be statistically significant.

### Identification of SL-responsive phosphoproteins

**Figure 4:**
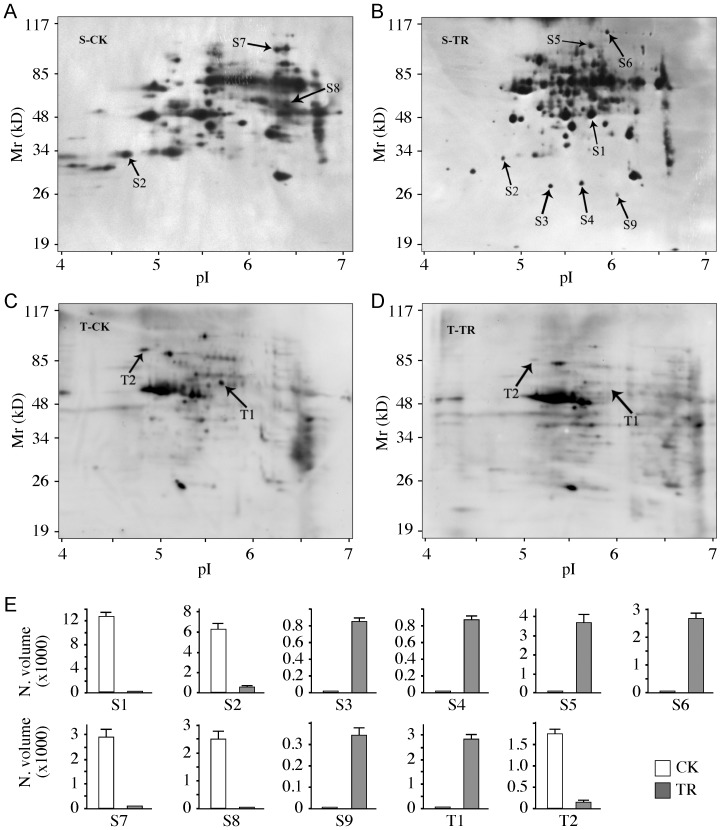
Phosphoproteins of *d10* seedlings in response to GR24 treatment. Total proteins were extracted from 6 day-old dark-grown *d10* mutant (XJC) seedlings in the absence (CK, the control) or presence of 1.0 μM GR24 (TR), and probed with anti-phosphoserine (a–b) or anti-phosphothreonine (c–d) antibodies. Phosphoproteins that changed phosphorylation intensities in response to GR24 treatment are indicated by S1–S9 and T1–T2. Intensity changes of phosphorylation status were quantified with the PDQuest 2D analysis software (e). Normalized volumes (N. volume x 1000) of phosphoprotein spots in the control (CK) or GR24 treated samples (TR) were presented.

Phosphoserine- and phosphothreonine-specific antibodies have been used to identify and characterize phosphoproteins via immunoblotting analysis in rice [47], [48]. In our research, total proteins were extracted from 6 day-old XJC seedlings that were grown in darkness in the presence of 1.0 μM GR24. Seedlings of the *d10* mutant XJC grown in the absence of GR24 served as a control. Proteins were resolved by 2-DE, and probed with antibodies against phosphoserine (pS) or phosphothreonine (pT). A total of 9 proteins (spots S1 to S9) reacted with the pS antibody exhibited differential phosphorylation intensity between the control and GR24 treatment (Figure 4A–B). Two pT-positive proteins (spots T1 and T2) showed differential phosphorylation intensity between the control and GR24 (Figure 4C–D). As shown in Figure 4E and Table 2, three differential patterns of phosphorylation intensity were distinguished: induced phosphorylation by GR24 treatment (S1、; S3、 S4、 S5、 S6 and S9), induced de-phosphorylation by GR24 (S8 and T1) and reduced phosphorylation intensity by GR24 (S2, S7 and T1). A total of eight proteins were identified by mass spectrometry from seven spots (Table 2). Spot T1 were identified as a mixture of two proteins. Disappointedly, the identities of four phosphoprotein spots (S2, S7, S9 and T2) was not made successfully by mass spectrometry largely due to their low abundance (Table 2). Among the identified phosphoproteins, phosphoglycerate kinase (PGK, spot S1), triosephosphate isomerase (TPI, spot S3), phosphomannose mutase (PMM, spot S4), NADH:ubiquinone oxidoreductase (Complex I, spot S5) and cytoplasmic aconitate hydratase (ACO, spot S6) were found to be phosphorylated in the *d10* mutant XJC seedlings induced by GR24 treatment (Figure 4A–B, Table 2). The aminotransferase AGD2 (AGD2, spot S8), and methylmalonate semi-aldehyde dehydrogenase (MSAH) and UDP-glucose pyrophosphorylase (UGP) of spot T11 were found to be dephosphorylated in the *d10* mutant XJC seedlings after treatment with GR24 (Figure 4, Table 2).

**Table 2:** Identification of proteins that changed phosphorylation intensities in response to SL treatment^a^.

Spot	GenBank Acc.	Protein name (abbreviation)	Mr(Da)/pI^b^	MP^c^	SC (%)^d^	Mowse score^e^	Exp. score (*P*-value)^f^	Intensity change^g^	FC±SE^h^	*p*-value^i^
S1	Q6H6C7	Phosphoglycerate kinase (PGK)	42196/5.6	15	43	97	1.4E-05	+		
S2		No identification						↓	10.2±0.5	0.0028
S3	Q8LR75	Triosephdosphate isomerase (TPI)	27484/5.6	8	32	68	0.01	+		
S4	Q7XPW5	Phosphomannose mutase (PMM)	28403/5.5	6	26	53	0.35	+		
S5	Q8W317	Mitochondrial NADH:ubiquinone oxidoreductase (Complex I)	82148/5.9	46	54	296	1.7E-25	+		
S6	Q6YZX6	Cytosolic aconitate hydratase (ACO)	98591/5.7	44	43	314	2.6E-27	+		
S7		No identification						↓	29.6±2.3	0.0032
S8	Q6VMN8	AGD2-like aminotransferase	50240/8.3	20	45	146	1.7E-10	-		
S9		No identification						+		
T1 (mix)	Q6Z4E4	(1) Methylmalonate semi-aldehyde dehydrogenase (MSDH)	57666/6.0	22	35	104	2.6E-06	-		
	Q93X08	(2) UDP-glucose pyrophosphorylase (UGP)	51821/5.4	20	40	95	2E-05	-		
T2		No identification						↓	10.5±0.5	0.0013

a Rice *d10* mutant XJC seedlings were germinated and grown on medium in the presence of 1.0 μM GR24 for 6 days in darkness. Seedlings grown without exogenous application of GR24 served as a control. Total proteins were extracted from seedlings and resolved by 2-DE. Antibodies against phosphoserine or phosphothreonine were used to probe the proteomes. Phosphoproteins that exhibited differences in intensity between GR24 treatment and the control were excised for identification by mass spectrometry.

b Relative molecular mass (Mr) in Da and isoelectric point (pI) of proteins are theoretical.

c Number of matched peptides (MP).

d Sequence coverage (SC, %) of the identified peptides.

e Proteins with a Mowse score of more than 61 were considered to be identified at a statistically significant level. Significance threshold *p*<0.05.

f Expectation scores are the probability-based values obtained from database search by MASCOT. Values less than their threshold (*p*<0.05) are statistically confident with more than 95% certainty.

g Protein phosphorylation intensity changes were distinguished as induced phosphorylation (+), induced de-phosphorylation (-) and reduced phosphorylation (↓) in response to GR24 treatment.

h Fold changes (FC) with standard errors (±SE) from three biological replicates were calculated as the ratio of normalized spot intensities between the treatment and control.

i Fold changes (FC) in spot intensity with a *p*-value of less than 0.05 were considered to be statistically significant.

## Discussion

### Inhibition of mesocotyl elongation of dark-grown *d10* seedlings by SLs

We observed that mesocotyl elongation in darkness was greater in the *d10* mutant than in the wild-type GC13. Exogenous application of GR24 restored the normal short mesocotyl phenotype in the *d10* mutant XJC seedlings grown in darkness, which is consistent with the previous observation by Hu et al [38], showing that SLs negatively regulate cell division, but not cell elongation, in the mesocotyl during germination and growth of rice in darkness. This negative regulation mechanism of SLs in mesocotyl development might be related to the role of SLs in the axillary buds, and might explain the observations that shoot branching of the *d* mutants is enhanced. To understand the general roles of SLs in mesocotyl elongation and axillary branching, we applied the proteomic approach to analyze the total protein and phosphoprotein profiles differentially expressed in dark-grown *d10* mutant XJC. A total of eight SL-responsive proteins were identified. They are mainly involved in carbohydrate metabolism, energy supply, defense responses, amino acid metabolism and cytoskeleton maintenance.

### Differential expression of key enzymes of metabolic pathways in response to SL treatment

Sucrose synthase 2 (SUS2, spot G1 Table 1) was the only protein that was up-regulated in dark-grown XJC seedlings by the application of GR24. The expression levels of all other differentially regulated proteins were down-regulated or even became undetectable. Sucrose synthase is a key enzyme in sucrose degradation and in directing sucrose to different metabolic pathways. Our observation was consistent with a previous report on the up-regulation of sucrose synthase 2 in *Arabidopsis* in response to BR treatment [44]. It is likely that sucrose synthase 2 is an important target of phytohormones.

The protein level of adenosine kinase (ADK, spot G4 Table 1) was down-regulated by GR24 application. ADK catalyzes the salvage synthesis of adenine monophosphate from adenosine and ATP. In *Arabidopsis*, the role of ADK in metabolism has been investigated in transgenic plants expressing either sense or antisense *ADK1* cDNA. Transgenic plants with less than 10% ADK activity show developmental abnormalities including reduced hypocotyl and root elongation [49]. ADK1 has also been shown to be involved in the development of root hairs and to negatively regulate systemic acquired resistance (SAR) via the jasmonic acid (JA) signaling pathway. We speculate that ADK may play a role as an inhibitor in the regulation of SL-related physiologic responses in rice.

Actin 7 (ACT7) is an important cytoskeleton protein in plants. Its differential expression has been found in the root proteome in rice treated with salt [50]. The down-regulated expression of ACT7 protein (spot G6, Figure 2) might be related the inhibitory effect of SLs on cell growth.

Vitamin B12-independent methionine synthase (MetE) is required for the biosynthesis of methionine and tetrahydrofolate from homocysteine and 5-methyl tetrahydrofolate [51], [52]. Methionine is an essential amino acid for protein biosynthesis and is also a precursor for *S*-adenosyl methionine (SAM), a methyl donor of various methylation reactions in cells. Under oxidative and temperature stress conditions, MetE is inactivated in bacteria and algae [53]. Our data showed that MetE expression level was down-regulated by GR24 (Figure 2, Table 1). It appears that MetE may represent a target of stress and phytohormones.

The G3 spot that was undetectable after GR24 treatment was identified as the hypothetical protein OsI_09330, which is homologous to maize 26S proteasome regulatory subunit 6A, a highly conserved protein in plants. It belongs to the P-loop nucleoside triphosphatase (NTPase) superfamily. By turning off the expression of the proteasome subunit, SLs may target the ubiquitin-mediated protein degradation machinery.

Late embryogenesis abundant (LEA) protein may protect plant cells from damages caused by stress [54]. The expression of LEA is known to be regulated by ABA, drought and wound [55]. *L*-Ascorbate peroxidase 2 (APX2) is an important antioxidant enzyme in protecting cells from oxidative stress. A previous report identifies APX2 as a differentially expressed protein in etiolated shoots of rice [56]. This is consistent with our observation that APX2 was found to be high in dark-grown rice seedlings, and was down-regulated in *d10* mutant plants in response to GR24. Because both LEA (spot 4) and APX2 (spot G8, Figure 2) were down-regulated by GR24, it appears that SLs may regulate plant growth and development through regulation of enzymes that function in defense against oxidative stress.

Mitochondrial phosphate translocator (MPT, spot G10) was found to be highly expressed in the dark-grown *d10* mutant seedlings, but became undetectable upon GR24 treatment (Figure 3, Table 1). This differential expression might be related to the inhibitory effect of GR24 on cell division. MPT is present as a homodimer in the inner mitochondrial membrane and transports inorganic phosphate (Pi) to the mitochondrial matrix. It is a symporter of phosphate (H_2_PO_4_^−^) and proton (H^+^) and plays an essential role in the oxidative phosphorylation from ADP to ATP in mitochondria [57]–[59]. A recent report has suggested that elevated SL levels by phosphate starvation contribute to the inhibition of tiller bud outgrowth in rice seedlings [60]. It is speculated that during phosphate deficiency, SLs may function as a rhizosphere signal to maximize AM fungi symbiosis for improved phosphate acquisition and may also serve as an endogenous hormone to optimize shoot branching for efficient Pi utilization. Our data showed that MTP disappeared in *d10* seedlings after SL treatment (Figure 3). This suggests that SLs may have a more complex regulation role in the Pi-related metabolism and cell division control in rice.

### Potential targets of the SL-signaling pathway

Protein phosphorylation has been recognized as an important mechanism for cell signaling and regulation of cell division and plant growth [61]. A great number of phosphoproteins have been implicated in the signaling pathways of phytohormones including GA, ABA and BR [42]–[44], [47], [62]. SLs represent a newly identified phytohormone. Whether phosphoproteins are involved in the signaling pathway of SLs remain to be explored. A total of 11 phosphoprotein spots were found to be responsive to GR24 treatment (Figure 4, Table 2). Eight proteins were identified by MALDI-TOF/MS, including six phosphoserine-containing proteins and two phosphothreonine-containing proteins. Unfortunately, four phosphoprotein spots (S2, S7, S9 and T2) were not identified successfully by mass spectrometry probably due to their low abundance. The signal intensity on X-ray films of Western blotting analysis using anti-phosphoserine/threonine antibodies was proportional to the phosphorylation levels of a protein, but not to the protein content. Despite of the high intensity on Western blots, these four phosphoprotein spots were faint on 2-DE gels (Figure 4). As the mass spectrometry technology continues to improve, these relatively low abundant proteins may be identified in future work.

Phosphoglycerate kinase (PGK) and triosephosphate isomerase (TPI) both are key enzymes in glycolysis. PGK catalyzes the conversion of 1,3-diphosphoglycerate to 3-phosphoglycerate, coupling with substrate level phosphorylation of ADP to ATP. TPI catalyzes the inter-conversion between dihydroxyacetone phosphate (DHAP) and 3-phosphoglyceraldehyde (PGA) in the glycolytic pathway. Phosphorylation of TPI has been found in the proteomes of rice leaves treated with exogenous ABA [47]. This suggests that TPI may be a target of phytohormone signaling. Our result is also consistent with a recent report on phosphoproteins in rice responsive to different hormones/stresses, which has concluded that the glycolytic pathway enzymes are the main targets of phosphorylation events [63].

Phosphomannose mutase (PMM), cytoplasmic aconitate hydratase (ACO), UDP-glucose pyrophosphorylase (UGP), and mitochondrial NADH:ubiquinone oxidoreductases (Complex I) are all important enzymes involved in the carbohydrate metabolism and ATP biosynthesis. PMM is the key enzyme in mannose metabolism and contributes to the biosynthesis of ascorbic acid,GDP mannose and mannosyl monophosphopolyprenol [64], [65]. ACO catalyzes the reversible isomerization between *cis*-aconitic acid and isocitric acid, and the cytoplasmic ACO has been implicated in multiple physiological processes including cytosolic citrate metabolism, the glyoxylate cycle, iron homeostasis and oxidative stress resistance. Complex I of the mitochondrial inner membrane consists of multiple subunits and provides a link of the tricarboxylic acid cycle with the oxidative phosphorylation. It uses the energy from the oxidation of NADH to produce reduced form of ubiquinone and generate a proton gradient that drives ATP biosynthesis.

UDP-glucose pyrophosphorylase (UGP) plays an important role in the synthesis of uridine diphosphoglucose (UDPG), which serves as the glucose donor for the biosynthesis of sucrose, cellulose, callose and cell wall hemicelluloses. Although the protein expression level of UGP did not change between control and GR24 treatment, the degree of de-phosphorylation of UGP was found to be responsive to GR24 (Figure 4, Table 2).

Aminotransferases transfer the amino group from an α-amino acid to an α-ketone acid, generating a new amino acid. A previous study has showed that the *aberrant growth and death* 2 (*agd2*) mutant of *Arabidopsis* has an elevated level of salicylic acid (SA), altered leaf morphology and mild dwarfism [66]. *Arabidopsis AGD2* encodes a novel aminotransferase whose physiological amino acid substrate(s) has yet to be defined [67]. The AGD2 aminotransferase appears to be involved in the synthesis of an essential amino acid-derived molecule affecting development and defense [67]. The roles of AGD2 phosphorylation changes in plant growth in response to SLs need further investigation.

Methylmalonate semialdehyde dehydrogenases (MSDH) belong to the CoA-dependent aldehyde dehydrogenase superfamily and catalyze the irreversible oxidative decarboxylation reaction of methylmalonate semialdehyde, generating NADH and propanoyl-CoA. MSDH may participate in the pathways of inositol, amino acid and propanoate metabolism. How the phosphorylation changes of MMDSH is related to branching and mesocotyl elongation in response to SL treatment remains to be investigated.

Understanding protein phosphorylation and its regulation is a crucial part of functional biology of plants. To investigate whether the eight SL-regulated phosphoproteins contain a conserved phosphorylation-site, we analyzed these proteins using Phosphoprotein BLAST in P^3^DB (http://www.p3db.org/) [68]. Except for S4 (Q7XPW5, phosphomannose mutase), other seven rice proteins have at least one orthologous phosphoprotein in *Arabidopsis thaliana*, *Medicago truncatula* and *Vitis vinifera* identified by different research groups [69]–[76]. Among them, S1 (Q6H6C7, phosphoglycerate kinase) and T1(1) (Q6Z4E4, methylmalonate semi-aldehyde dehydrogenase) have been identified as phosphoproteins by large-scale comparative phosphoproteomics in rice [69]. Eight orthologous accessions of S1 in the database include four from *Oryza sativa*, one from *Arabidopsis thaliana* and three from *Medicago truncatula*. The phosphorylation site of S1 (Q6H6C7) has been identified as serine-201 in rice [69].

It has recently been shown that depletion of the auxin efflux carrier PIN1 from the plasma membrane occurs within 10 minutes after SL treatment and does not depend upon protein synthesis [77]. This suggests that a post-translational regulatory mechanism, rather than a transcriptional cascade, may carry out the initial responses to SL perception. As an important post-translational modification, protein phosphorylation and de-phosphorylation might have rapid effects on enzyme activity, structural changes and subcellular localization. Thus protein phosphorylation status might be an important target of SL action.

Despite of the successful identification of phosphorylation changes of several proteins, some known SL-related signaling components such as D3 (an F-box protein) and D14 (an α/β-fold hydrolase) were not detected in our study. It is possible that they are present in low abundance or the limited pH range of IPG strips are not suitable for their identification.

D3/MAX2 encodes an F-box protein that is highly conserved among land plants [78], [79]. F-box proteins serve as the adapter components of SCF E3 ubiquitin ligase complexes, and confer substrate specificity for ubiquitination. Target proteins recognized by the F-box protein are typically polyubiquitinated and subsequently degraded by the 26S proteasome [80]. There are remarkable similarities in protein components between the SL and gibberellin signaling pathways. Both the gibberellin receptor and the putative SL receptor D14 are members of the α/β hydrolase protein family, and in both cases, the hormone signaling requires the function of an F-box protein [81]–[83]. Two recent reports on rice D53, a member of the double Clp-N motif-containing P-loop nucleoside triphosphate hydrolase superfamily, further reveal similarities between the signaling mechanisms between SL and gibberellins [84], [85]. D53 functions as a repressor of SL signaling and a target of D3/MAX2. Perception of SL by D14 and the SCF^D3^ complex leads to ubiquitination of D53 and its subsequent degradation by the ubiquitin proteasome system, which in turn releases the repression of downstream target genes [84], [85]. As an important component of the ubiquitin proteasome system, 26S proteasome regulatory subunit 6A (spot G3) was identified in this study as a differentially expressed protein in response to GR24 treatment. We speculate that it might play a role in the ubiquitin-mediated protein degradation machinery in the SL signaling pathway. It remains to be determined whether this 26S proteasome regulatory subunit interacts with the D3-D14-D53 complex, and if it plays a role in the ubiquitin-mediated protein degradation machinery in the SL signaling pathway.

In summary, the 8 differentially expressed proteins responded to SLs did not overlap with the 8 proteins that exhibited phosphorylation status changes. Thus, different approaches have apparent different sensitivities and specificities in the identification of proteins in response to SL treatment. An integrative approach to combine the use of multiple techniques may complement with each other and lead to better identification of molecular changes in rice plants. It will be of interest to determine the exact functions of the SL-regulated proteins identified in this work. Due to the inherent bias of the 2-DE approach toward the identification of more abundant proteins, components of signaling pathways with lower abundance would be more difficult to detect. Enrichment of these low abundant proteins using pre-fractionation methods and application of more sensitive technologies will be helpful. This work represented an attempt to use proteomic approach in identification of proteins that responded to SL treatment in rice, and may pave an avenue for future investigations aimed at exploration of new signaling pathways in plants.

## Materials and Methods

### Plant materials and growth conditions

Rice (*Oryza sativa* L. ssp. *indica*) cultivar GC13 (the wild type) and its *d10* mutant line XJC (*d10*) were used in this research. XJC was originally isolated from a mutation population generated by γ-ray irradiation. After inbreeding for several generations, XJC attained genetic stability and exhibited semi-dwarf plant height and a high-tillering phenotype. More than 120 tillers could be observed in XJC at the maturing stage [46].

For the phenotype rescuing experiment, pregerminated rice seeds were grown hydroponically in growth chambers and cultured at 30°C under fluorescence white light with a 16 h light/8 h dark photoperiod for two weeks. The seedlings were maintained in the Kimura B hydroponic solution [86].

For the mesocotyl elongation experiment, rice seedlings were grown under conditions as described elsewhere [38] with minor modifications. Dehusked rice caryopses were sterilized in a 10% (v/v) sodium hypochlorite solution for 1 h. After washing with deionized water, seeds were kept in water for 12 h at room temperature in darkness. Each agar plate (0.7%, w/v) was planted with 6 seeds, and placed in a box covered by black cloths. The box was kept in a growth chamber at 30°C for 6 days.

### GR24 treatment and analysis

GR24, kindly provided by Prof. S. Yamaguchi, was dissolved in 100% acetone as a 100 mM stock solution, and was added to the hydroponic culture medium in a final concentration of 1.0 μM. Hydroponic culture medium with 0.001% acetone served as a negative control. For the mesocotyl elongation experiment, GR24 (100 mM stock solution in acetone) was added to melted 0.7% (w/v) agar medium (about 50°C) in a final concentration of 1.0 μM GR24. Acetone at a final concentration of 0.001% was present in each agar plate including the mock treatment. Sterilized seeds were placed on agar plates and seedlings were grown at 30°C for 6 days under complete darkness. The mesocotyl length of wild type GC13 and *d10* mutant XJC seedlings were recorded. Total proteins were extracted from XJC seedlings with or without GR24 treatment, and analyzed by the proteomic approach.

### Preparation of total proteins

Etiolated XJC seedlings, cleared off residual seeds, were quickly frozen in liquid nitrogen and stored at −80°C. Total proteins were extracted in extraction buffer and precipitated by trichloroacetic acid as described previously [87]. Protein pellets were dissolved in a final concentration of approximately 25 mg/ml protein in the O'Farrells UKS buffer (9.5 M urea, 5 mM K_2_CO_3_, 1.25% sodium dodecyl sulfate (SDS), 0.5% DTT, pH 3.5 to 10.0 2% pharmalyte, 6% Triton X-100) [39]. To facilitate protein dissolution, the pellet-buffer mixture was shaken gently at room temperature for 1 h, followed by centrifugation at 15,000 g at 15°C for 30 min. Protein content of the soluble fraction was determined using the BCA Protein Assay (Pierce). The soluble fraction was used for two-dimensional polyacrylmide gel electrophoresis (2-DE) or stored at −80°C. Because most of the proteins were found within pI 4–7 on 2D gels (Supplemental Figure S1), precast immobilized pH gradient (IPG) strips (pH 4–7, 7 cm, Bio-Rad) were used in the first dimension of electrophoresis. Proteins (approximately 100 μg) were loaded on each strip. After isoelectric focusing electrophoresis, the IPG strips were soaked in equilibration buffer and placed directly onto 12% polyacrylamide-SDS slab gels. After electrophoresis, the gels were immuno-blotted using a phosphor-amino acid-specific antibody. Identical gels were also stained by Coomassie blue or silver staining solution.

### Immunoblot analysis

Proteins resolved on 2-DE gels were transferred onto PVDF membranes (Millipore). The membranes were blocked with 5% (w/v) bovine serum albumin (BSA) in TBST (20 mM Tris–HCl, pH 7.5, 150 mM NaCl, 0.05% (v/v) Tween-20) for overnight at 4°C, and then incubated with mouse anti-phosphoserine (Millipore) or rabbit anti-phosphothreonine antibodies (Cell Signaling) at 1 μg/ml in TBST for 1.5 hr. After washing with TBST, membranes were incubated with goat anti-mouse IgG or donkey anti-rabbit IgG (Protein Tech Group) conjugated with horseradish peroxidase at a 1∶4000 dilution for 1 h at room temperature. Immunoreactive spots were detected by enhanced chemiluminescence (GE) followed by exposure to X-ray film according to the manufacturer's instructions.

### Image acquisition and data analysis

Stained gels and developed films were scanned with the Molecular Imager ChemiDoc XRS System (Bio-Rad, USA) in transmission mode. All 2-DE gel images were captured, digitalized, and analyzed with PDQuest software (Bio-Rad) using tenfold over background as a minimum criterion for presence/absence. The analysis was re-evaluated by visual inspection. The spots present in all three biological replicates showing statistically significant, qualitative or quantitative differences between treatment (TR) and control (CK) were drawn after comparisons. On the basis of total quantity and intensity of valid spots in each 2D-gel, a normalization factor was generated. A normalized spot volume was determined for each protein spot. Data analysis was performed with Excel software (Microsoft). Error bars shown in figures represent the standard deviation values.

### Mass spectrometry analysis and database search

Selected protein spots were cut from the gel and digested with sequencing-grade trypsin (Promega). The peptides were dissolved in 2 μL of 0.5% trifluoroacetic acid (TFA) solution and mixed with an equal volume of a saturated matrix solution consisting of α-cyano-4-hydroxy-cinnamic acid in 70% acetonitrile with 0.1% TFA. The resulting mixture was loaded onto the point template. After drying at room temperature, the template was introduced into a Bruker ReflexTM matrix-assisted laser-desorption ionization-time of flight mass spectrometry (MALDI-TOF MS) system (Bruker, Germany). The peak lists of peptide mass fingerprints were created using the FlexAnalysis 2.0 software (Bruker Daltonics), and interpreted with MASCOT (Matrix Science, London, UK. http://www.matrixscience.com/) against the MSDB and NCBI databases.

## Supporting Information

Figure S1 **Comparison of protein distribution patterns separated by different precast immobilized pH gradient (IPG) strips.** Total proteins isolated from rice seedlings were separated using IPG strips with a pH range of 3–10 (left) and pH 4–7 (right). Note that the majority of proteins were present within the pH range of 4–7.
(PDF)

## References

[1] Gomez-Roldan V, Fermas S, Brewer PB, Puech-Pages V, Dun EA, et al (2008) Strigolactone inhibition of shoot branching. Nature 455: 189–194. 18690209 10.1038/nature07271

[2] Umehara M, Hanada A, Yoshida S, Akiyama K, Arite T, et al (2008) Inhibition of shoot branching by new terpenoid plant hormones. Nature 455: 195–200. 18690207 10.1038/nature07272

[3] Ishikawa S, Maekawa M, Arite T, Onishi K, Takamure I, et al (2005) Suppression of tiller bud activity in tillering dwarf mutants of rice. Plant Cell Physiol 46: 79–86. 15659436 10.1093/pcp/pci022

[4] Arite T, Iwata H, Ohshima K, Maekawa M, Nakajima M, et al (2007) *DWARF10*, an *RMS1/MAX4/DAD1* ortholog, controls lateral bud outgrowth in rice. Plant J 51: 1019–1029. 17655651 10.1111/j.1365-313X.2007.03210.x

[5] Zou J, Zhang S, Zhang W, Li G, Chen Z, et al (2006) The rice *HIGH-TILLERING DWARF1* encoding an ortholog of *Arabidopsis* MAX3 is required for negative regulation of the outgrowth of axillary buds. Plant J 48: 687–698. 17092317 10.1111/j.1365-313X.2006.02916.x

[6] Wang Y, Li J (2008) Molecular basis of plant architecture. Annu Rev Plant Biol 59: 253–279. 18444901 10.1146/annurev.arplant.59.032607.092902

[7] Beveridge CA, Ross JJ, Murfet IC (1994) Branching mutant *rms-2* in *Pisum sativum* (Grafting studies and endogenous indole-3-acetic acid levels). Plant Physiol 104: 953–959. 12232140 10.1104/pp.104.3.953 PMC160693

[8] Beveridge CA, Ross JJ, Murfet IC (1996) Branching in pea (action of genes *Rms3* and *Rms4*). Plant Physiol 110: 859–865. 12226224 10.1104/pp.110.3.859 PMC157785

[9] Napoli C (1996) Highly branched phenotype of the petunia dad1-1 mutant is reversed by grafting. Plant Physiol 111: 27–37. 12226274 10.1104/pp.111.1.27 PMC157810

[10] Morris SE, Turnbull CG, Murfet IC, Beveridge CA (2001) Mutational analysis of branching in pea. Evidence that Rms1 and Rms5 regulate the same novel signal. Plant Physiol 126: 1205–1213. 11457970 10.1104/pp.126.3.1205 PMC116476

[11] Stirnberg P, van De Sande K, Leyser HMO (2002) *MAX1* and *MAX2* control shoot lateral branching in *Arabidopsis* . Development 129: 1131–1141. 11874909 10.1242/dev.129.5.1131

[12] Sorefan K, Booker J, Haurogne K, Goussot M, Bainbridge K, et al (2003) *MAX4* and *RMS1* are orthologous dioxygenase-like genes that regulate shoot branching in *Arabidopsis* and pea. Genes Dev 17: 1469–1474. 12815068 10.1101/gad.256603 PMC196077

[13] Snowden KC, Simkin AJ, Janssen BJ, Templeton KR, Loucas HM, et al (2005) The *decreased apical dominance1*/*Petunia hybrida CAROTENOID CLEAVAGE DIOXYGENASE8* gene affects branch production and plays a role in leaf senescence, root growth, and flower development. Plant Cell 17: 746–759. 15705953 10.1105/tpc.104.027714 PMC1069696

[14] Zou J, Chen Z, Zhang S, Zhang W, Jiang G, et al (2005) Characterizations and fine mapping of a mutant gene for high tillering and dwarf in rice (*Oryza sativa* L.). Planta 222: 604–612. 16021500 10.1007/s00425-005-0007-0

[15] Arite T, Umehara M, Ishikawa S, Hanada A, Maekawa M, et al (2009) *d14*, a strigolactone-insensitive mutant of rice, shows an accelerated outgrowth of tillers. Plant Cell Physiol 50: 1416–1424. 19542179 10.1093/pcp/pcp091

[16] Simons JL, Napoli CA, Janssen BJ, Plummer KM, Snowden KC (2007) Analysis of the *DECREASED APICAL DOMINANCE* genes of petunia in the control of axillary branching. Plant Physiol 143: 697–706. 17158589 10.1104/pp.106.087957 PMC1803742

[17] Gao Z, Qian Q, Liu X, Yan M, Feng Q, et al (2009) *Dwarf 88*, a novel putative esterase gene affecting architecture of rice plant. Plant Mol Biol 71: 265–276. 19603144 10.1007/s11103-009-9522-x

[18] Lin H, Wang R, Qian Q, Yan M, Meng X, et al (2009) *DWARF27*, an iron-containing protein required for the biosynthesis of strigolactones, regulates rice tiller bud outgrowth. Plant Cell 21: 1512–1525. 19470589 10.1105/tpc.109.065987 PMC2700539

[19] Liu W, Wu C, Fu Y, Hu G, Si H, et al (2009) Identification and characterization of *HTD2*: a novel gene negatively regulating tiller bud outgrowth in rice. Planta 23: 649–658. 10.1007/s00425-009-0975-6 19579033

[20] Booker J, Auldridge M, Wills S, McCarty D, Klee H, et al (2004) MAX3/CCD7 is a carotenoid cleavage dioxygenase required for the synthesis of a novel plant signaling molecule. Curr Biol 14: 1232–1238. 15268852 10.1016/j.cub.2004.06.061

[21] Alder A, Jamil M, Marzorati M, Bruno M, Vermathen M, et al (2012) The path from β-carotene to carlactone, a strigolactone-like plant hormone. Science 335: 1348–1351. 22422982 10.1126/science.1218094

[22] Scaffidi A, Waters MT, Ghisalberti EL, Dixon KW, Flematti GR, et al (2013) Carlactone-independent seedling morphogenesis in *Arabidopsis* . Plant J 76: 1–9. 23773129 10.1111/tpj.12265

[23] Zhao LH, Zhou XE, Wu ZS, Yi W, Xu Y, et al (2013) Crystal structures of two phytohormone signal-transducing α/β hydrolases: karrikin-signaling KAI2 and strigolactonesignaling DWARF14. Cell Res 23: 436–439. 23381136 10.1038/cr.2013.19 PMC3587710

[24] Kagiyama M, Hirano Y, Mori T, Kim SY, Kyozuka J, et al (2013) Structures of D14 and D14L in the strigolactone and karrikin signaling pathways. Genes Cells 18: 147–160. 23301669 10.1111/gtc.12025

[25] Hamiaux C, Drummond RS, Janssen BJ, Ledger SE, Cooney JM, et al (2012) DAD2 is an α/β hydrolase likely to be involved in the perception of the plant branching hormone, strigolactone. Curr Biol 22: 1–5. 22959345 10.1016/j.cub.2012.08.007

[26] Yamaguchi S, Kyozuka J (2010) Branching hormone is busy both underground and overground. Plant Cell Physiol 51: 1091–1094. 20621958 10.1093/pcp/pcq088

[27] Cardoso C, Ruyter-Spira C, Bouwmeester HJ (2011) Strigolactones and root infestation by plant-parasitic *Striga*, *Orobanche* and *Phelipanche* spp. Plant Sci 180: 414–420. 21421387 10.1016/j.plantsci.2010.11.007

[28] Bouwmeester HJ, Matusova R, Zhongkui S, Beale MH (2003) Secondary metabolite signalling in host–parasitic plant interactions. Curr Opin Plant Biol 6: 358–364. 12873531 10.1016/s1369-5266(03)00065-7

[29] Ruyter-Spira C, Al-Babili S, van der Krol S, Bouwmeester H (2013) The biology of strigolactones. Trends Plant Sci 18: 72–83. 23182342 10.1016/j.tplants.2012.10.003

[30] Mayzlish-Gati E, De Cuyper C, Goormachtig S, Beeckman T, Vuylsteke M, et al (2012) Strigolactones are involved in root response to low phosphate conditions in *Arabidopsis* . Plant Physiol 160: 1329–1341. 22968830 10.1104/pp.112.202358 PMC3490576

[31] Rasmussen A, Mason MG, De Cuyper C, Brewer PB, Herold S, et al (2012) Strigolactones suppress adventitious rooting in *Arabidopsis* and pea. Plant Physiol 158: 1976–1987. 22323776 10.1104/pp.111.187104 PMC3320200

[32] Yan H, Saika H, Maekawa M, Takamure I, Tsutsumi N, et al (2007) Rice tillering dwarf mutant *dwarf3* has increased leaf longevity during darkness-induced senescence or hydrogen peroxide-induced cell death. Genes Genet Syst 82: 361–366. 17895586 10.1266/ggs.82.361

[33] Mashiguchi K, Sasaki E, Shimada Y, Nagae M, Ueno K, et al (2009) Feedback-regulation of strigolactone biosynthetic genes and strigolactone-regulated genes in *Arabidopsis* . Biosci Biotechnol Biochem 73: 2460–2465. 19897913 10.1271/bbb.90443

[34] Jones AM, Cochran DS, Lamerson PM, Evans ML, Cohen JD (1991) Red light-regulated growth: 1. Changes in the abundance of indoleacetic acid and a 22-kilodalton auxin-binding protein in the maize mesocotyl. Plant Physiol 97: 352–358. 11538374 10.1104/pp.97.1.352 PMC1081005

[35] Sawers RJ, Linley PJ, Farmer PR, Hanley NP, Costich DE, et al (2002) *elongated mesocotyl1*, a phytochrome-deficient mutant of maize. Plant Physiol 130: 155–163. 12226496 10.1104/pp.006411 PMC166549

[36] Mori M, Nomura T, Ooka H, Ishizaka M, Yokota T, et al (2002) Isolation and characterization of a rice dwarf mutant with a defect in brassinosteroid biosynthesis. Plant Physiol 130: 1152–1161. 12427982 10.1104/pp.007179 PMC166636

[37] Choi D, Lee Y, Cho H, Kende H (2003) Regulation of expansin gene expression affects growth and development in transgenic rice plants. Plant Cell 15: 1386–1398. 12782731 10.1105/tpc.011965 PMC156374

[38] Hu Z, Yan H, Yang J, Yamaguchi S, Maekawa M, et al (2010) Strigolactones negatively regulate mesocotyl elongation in rice during germination and growth in darkness. Plant Cell Physiol 51: 1136–1142. 20498118 10.1093/pcp/pcq075 PMC2900821

[39] O'Farrell PH (1975) High resolution two-dimensional electrophoresis of proteins. J Biol Chem 250: 4007–4021. 236308 PMC2874754

[40] Unlu M, Morgan ME, Minden JS (1997) Difference gel electrophoresis: a single gel method for detecting changes in protein extracts. Electrophoresis 18: 2071–2077. 9420172 10.1002/elps.1150181133

[41] Lewis TS, Hunt JB, Aveline LD, Jonscher KR, Louie DF, et al (2000) Identification of novel MAP kinase pathway signaling targets by functional proteomics and mass spectrometry. Mol Cell 6: 1343–1354. 11163208 10.1016/s1097-2765(00)00132-5

[42] Deng Z, Zhang X, Tang W, Oses-Prieto JA, Suzuki N, et al (2007) A proteomics study of brassinosteroid response in *Arabidopsis* . Mol Cell Proteomics 6: 2058–2071. 17848588 10.1074/mcp.M700123-MCP200 PMC2966871

[43] Tang W, Deng Z, Oses-Prieto JA, Suzuki N, Zhu S, et al (2008a) Proteomics studies of brassinosteroid signal transduction using prefractionation and two-dimensional DIGE. Mol Cell Proteomics 7: 728–738. 18182375 10.1074/mcp.M700358-MCP200 PMC2401332

[44] Tang W, Kim TW, Oses-Prieto JA, Sun Y, Deng Z, et al (2008b) BSKs mediate signal transduction from the receptor kinase BRI1 in *Arabidopsis* . Science 321: 557–560. 18653891 10.1126/science.1156973 PMC2730546

[45] Kaufmann H, Bailey JE, Fussenegger M (2001) Use of antibodies for detection of phosphorylated proteins separated by two-dimensional gel electrophoresis. Proteomics 1: 194–199. 11680866 10.1002/1615-9861(200102)1:2<194::AID-PROT194>3.0.CO;2-K

[46] Chen F, Jiang L, Zheng J, Huang R, Wang H, et al (2013) Identification of a cosegregative protein with the tillering trait in rice (*Oryza sativa* L.). Plant Omics J 6: 36–45.

[47] He H, Li J (2008) Proteomic analysis of phosphoproteins regulated by abscisic acid in rice leaves. Biochem Biophys Res Commun 371: 883–888. 18468508 10.1016/j.bbrc.2008.05.001

[48] Ke Y, Han G, He H, Li J (2009) Differential regulation of proteins and phosphoproteins in rice under drought stress. Biochem Biophys Res Commun 379: 133–138. 19103168 10.1016/j.bbrc.2008.12.067

[49] Moffatt BA, Stevens YY, Allen MS, Snider JD, Pereira LA, et al (2002) Adenosine kinase deficiency is associated with developmental abnormalities and reduced transmethylation. Plant Physiol 128: 812–821. 11891238 10.1104/pp.010880 PMC152195

[50] Chitteti BR, Peng ZH (2007) Proteome and phosphoproteome differential expression under salinity stress in rice (*Oryza sativa*) roots. J Proteome Res 6: 1718–1727. 17385905 10.1021/pr060678z

[51] Roth JR, Lawrence JG, Bobik TA (1996) Cobalamin (coenzyme B12): synthesis and biological significance. Annu Rev Microbiol 50: 137–181. 8905078 10.1146/annurev.micro.50.1.137

[52] Warren MJ, Raux E, Schubert HL, Escalante-Semerena JC (2002) The biosynthesis of adenosylcobalamin (vitamin B12). Nat Prod Rep19: 390–412. 10.1039/b108967f 12195810

[53] Hondorp ER, Matthews RG (2004) Oxidative stress inactivates cobalamin-independent methionine synthase (MetE) in *Escherichia coli* . PLoS Biol 2: e336. 15502870 10.1371/journal.pbio.0020336 PMC521173

[54] Shinozaki K, Yamaguchi-Shinozaki K (2007) Gene networks involved in drought stress response and tolerance. J Exp Bot 58: 221–227. 17075077 10.1093/jxb/erl164

[55] Zegzouti H, Jones B, Marty C, Lelièvre JM, Latché A, et al (1997) ER5, a tomato cDNA encoding an ethylene-responsive LEA-like protein: characterization and expression in response to drought, ABA and wounding. Plant Mol Biol 35: 847–854. 9426604 10.1023/a:1005860302313

[56] Komatsu S, Muhammad A, Rakwal R (1999) Separation and characterization of proteins from green and etiolated shoots of rice (*Oryza sativa* L.): Towards a rice proteome. Electrophoresis 20: 630–636. 10217180 10.1002/(SICI)1522-2683(19990301)20:3<630::AID-ELPS630>3.0.CO;2-Z

[57] Capobianco L, Brandolin G, Palmieri F (1991) Transmembrane topography of the mitochondrial phosphate carrier explored by peptide-specific antibodies and enzymatic digestion. Biochem J 30: 4963–4969. 10.1021/bi00234a018 2036364

[58] Palmieri F, Bisaccia F, Capobianco L, Dolce V, Fiermonte G, et al (1993) Transmembrane topology, genes and biogenesis of the mitochondrial phosphate and oxoglutarate carriers. J Bioenerg Biomembr 25: 493–501. 8132489 10.1007/BF01108406

[59] Stappen R, Kramer R (1994) Kinetic mechanism of phosphate/phosphate and phosphate/OH-antiport catalyzed by reconstituted phosphate carrier from beef heart mitochondria. J Biol Chem 269: 11240–11246. 8157653

[60] Umehara M, Hanada A, Magome H, Takeda-Kamiya N, Yamaguchi S (2010) Contribution of strigolactones to the inhibition of tiller bud outgrowth under phosphate deficiency in rice. Plant Cell Physiol 51: 1118–1126. 20542891 10.1093/pcp/pcq084 PMC2900824

[61] Komatsu S, Hirano H (1993) Protein kinase activity and protein phosphorylation in rice (*Oryza sativa* L.) leaf. Plant Sci 94: 127–137.

[62] Gomi K, Matsuoka M (2003) Gibberellin signalling pathway. Curr Opin Plant Biol 6: 489–493. 12972050 10.1016/s1369-5266(03)00079-7

[63] Khan M, Jan A, Karibe H, Komatsu S (2005) Identification of phosphoproteins regulated by gibberellin in rice leaf sheath. Plant Mol Biol 58: 27–40. 16028114 10.1007/s11103-005-4013-1

[64] Qian W, Yu C, Qin H, Liu X, Zhang A, et al (2007) Molecular and functional analysis of phosphomannomutase (PMM) from higher plants and genetic evidence for the involvement of PMM in ascorbic acid biosynthesis in *Arabidopsis* and *Nicotiana benthamiana* . Plant J 49: 399–413. 17217471 10.1111/j.1365-313X.2006.02967.x

[65] Hoeberichts FA, Vaeck E, Kiddle G, Coppens E, van-de-Cotte B, et al (2008) A temperature-sensitive mutation in the *Arabidopsis thaliana* phosphomannomutase gene disrupts protein glycosylation and triggers cell death. J Biol Chem 283: 5708–5718. 18086684 10.1074/jbc.M704991200

[66] Rate DN, Greenberg JT (2001) The *Arabidopsis aberrant growth and death2* mutant shows resistance to *Pseudomonas syringae* and reveals a role for NPR1 in suppressing hypersensitive cell death. Plant J 27: 203–211. 11532166 10.1046/j.0960-7412.2001.1075umedoc.x

[67] Song JT, Lu H, Greenberg JT (2004) Divergent roles in *Arabidopsis thaliana* development and defense of two homologous genes, *ABERRANT GROWTH AND DEATH2* and *AGD2-LIKE DEFENSE RESPONSE PROTEIN1*, encoding novel aminotransferases. Plant Cell 16: 353–366. 14729919 10.1105/tpc.019372 PMC341909

[68] Gao J, Agrawal GK, Thelen JJ, Xu D (2009) P3DB: a plant protein phosphorylation database. Nucleic Acids Res 37: D960–D962. 18931372 10.1093/nar/gkn733 PMC2686431

[69] Nakagami H, Sugiyama N, Mochida K, Daudi A, Yoshida Y, et al (2010) Large-scale comparative phosphoproteomics identifies conserved phosphorylation sites in plants. Plant Physiol 153: 1161–1174. 20466843 10.1104/pp.110.157347 PMC2899915

[70] Sugiyama N, Nakagami H, Mochida K, Daudi A, Tomita M, et al (2008) Large-scale phosphorylation mapping reveals the extent of tyrosine phosphorylation in *Arabidopsis* . Mol Syst Biol 4: 193. 18463617 10.1038/msb.2008.32 PMC2424297

[71] Reiland S, Messerli G, Baerenfaller K, Gerrits B, Endler A, et al (2009) Large-scale *Arabidopsis* phosphoproteome profiling reveals novel chloroplast kinase substrates and phosphorylation networks. Plant Physiol 150: 889–903. 19376835 10.1104/pp.109.138677 PMC2689975

[72] Engelsberger WR, Schulze WX (2012) Nitrate and ammonium lead to distinct global dynamic phosphorylation patterns when resupplied to nitrogen-starved *Arabidopsis* seedlings. Plant J 69: 978–995. 22060019 10.1111/j.1365-313X.2011.04848.x PMC3380553

[73] Rose CM, Venkateshwaran M, Volkening JD, Grimsrud PA, Maeda J, et al (2012) Rapid phosphoproteomic and transcriptomic changes in the rhizobia-legume symbiosis. Mol Cell Proteomics 11: 724–744. 22683509 10.1074/mcp.M112.019208 PMC3434772

[74] Mayank P, Grossman J, Wuest S, Boisson-Dernier A, Roschitzki B, et al (2012) Characterization of the phosphoproteome of mature *Arabidopsis* pollen. Plant J 72: 89–101. 22631563 10.1111/j.1365-313X.2012.05061.x

[75] Grimsrud PA, den Os D, Wenger CD, Swaney DL, Schwartz D, et al (2010) Large-scale phosphoprotein analysis in *Medicago truncatula* roots provides insight into in vivo kinase activity in legumes. Plant Physiol 152: 19–28. 19923235 10.1104/pp.109.149625 PMC2799343

[76] Melo-Braga MN, Verano-Braga T, León IR, Antonacci D, Nogueira FC, et al (2012) Modulation of protein phosphorylation, N-glycosylation and Lys-acetylation in grape (*Vitis vinifera*) mesocarp and exocarp owing to *Lobesia botrana* infection. Mol Cell Proteomics 11: 945–956. 22778145 10.1074/mcp.M112.020214 PMC3494143

[77] Shinohara N, Taylor C, Leyser O (2013) Strigolactone can promote or inhibit shoot branching by triggering rapid depletion of the auxin efflux protein PIN1 from the plasma membrane. PLoS Biol 11: e1001474. 23382651 10.1371/journal.pbio.1001474 PMC3558495

[78] Delaux P, Xie X, Timme R, Peuech-Pages V, Dunand C, et al (2012) Origin of strigolactones in the green lineage. New Phytol 195: 857–871. 22738134 10.1111/j.1469-8137.2012.04209.x

[79] Waters MT, Smith SM, Nelson DC (2011) Smoke signals and seed dormancy: where next for MAX2? Plant Sig Behav 6: 1418–1422. 10.4161/psb.6.9.17303 PMC3258081 22019642

[80] Somers DE, Fujiwara S (2009) Thinking outside the F-box: novel ligands for novel receptors. Trends Plant Sci 14: 206–213. 19285909 10.1016/j.tplants.2009.01.003

[81] Sun TP (2011) The molecular mechanism and evolution of the GA-GID1-DELLA signaling module in plants. Curr Biol 21: R338–R345. 21549956 10.1016/j.cub.2011.02.036

[82] Nelson DC, Scaffidi A, Dun EA, Waters MT, Flematti GR, et al (2011) F-box protein MAX2 has dual roles in karrikin and strigolactone signaling in *Arabidopsis thaliana* . Proc Natl Acad Sci USA 108: 8897–8902. 21555559 10.1073/pnas.1100987108 PMC3102411

[83] Waters MT, Nelson DC, Scaffidi A, Flematti GR, Sun YK, et al (2012) Specialisation within the DWARF14 protein family confers distinct responses to karrikins and strigolactones in *Arabidopsis* . Development 139: 1285–1295. 22357928 10.1242/dev.074567

[84] Jiang L, Liu X, Xiong G, Liu H, Chen F, et al (2013) DWARF 53 acts as a repressor of strigolactone signalling in rice. Nature 504: 401–405. 24336200 10.1038/nature12870 PMC5802366

[85] Zhou F, Lin Q, Zhu L, Ren Y, Zhou K, et al (2013) D14-SCF^D3^-dependent degradation of D53 regulates strigolactone signaling. Nature 504: 406–410. 24336215 10.1038/nature12878 PMC4096652

[86] Hsu YT, Kao CH (2005) Abscisic acid accumulation and cadmium tolerance in rice seedlings. Physiol Plant 124: 71–80.

[87] Damerval C, de Vienne D, Zivy M, Thiellement H (1986) Technical improvements in two-dimensional electrophoresis increase the level of genetic-variation detected in wheat-seedling proteins. Electrophoresis 7: 52–54.

