# Supplementary material for: Identification of Differentially Expressed Proteins and Phosphorylated Proteins in Rice Seedlings in Response to Strigolactone Treatment
Source: PLoS One. 2014 Apr 3;9(4):e93947. doi: 10.1371/journal.pone.0093947 (PMC3974870; doi:10.1371/journal.pone.0093947)
Supplement: Figure S1 — Comparison of protein distribution patterns separated by different precast immobilized pH gradient (IPG) strips. Total proteins isolated from rice seedlings were separated using IPG strips with a pH range of 3–10 (left) and pH 4–7 (right). Note that the majority of proteins were present within the pH range of 4–7. (PDF) [file pone.0093947.s001.pdf]

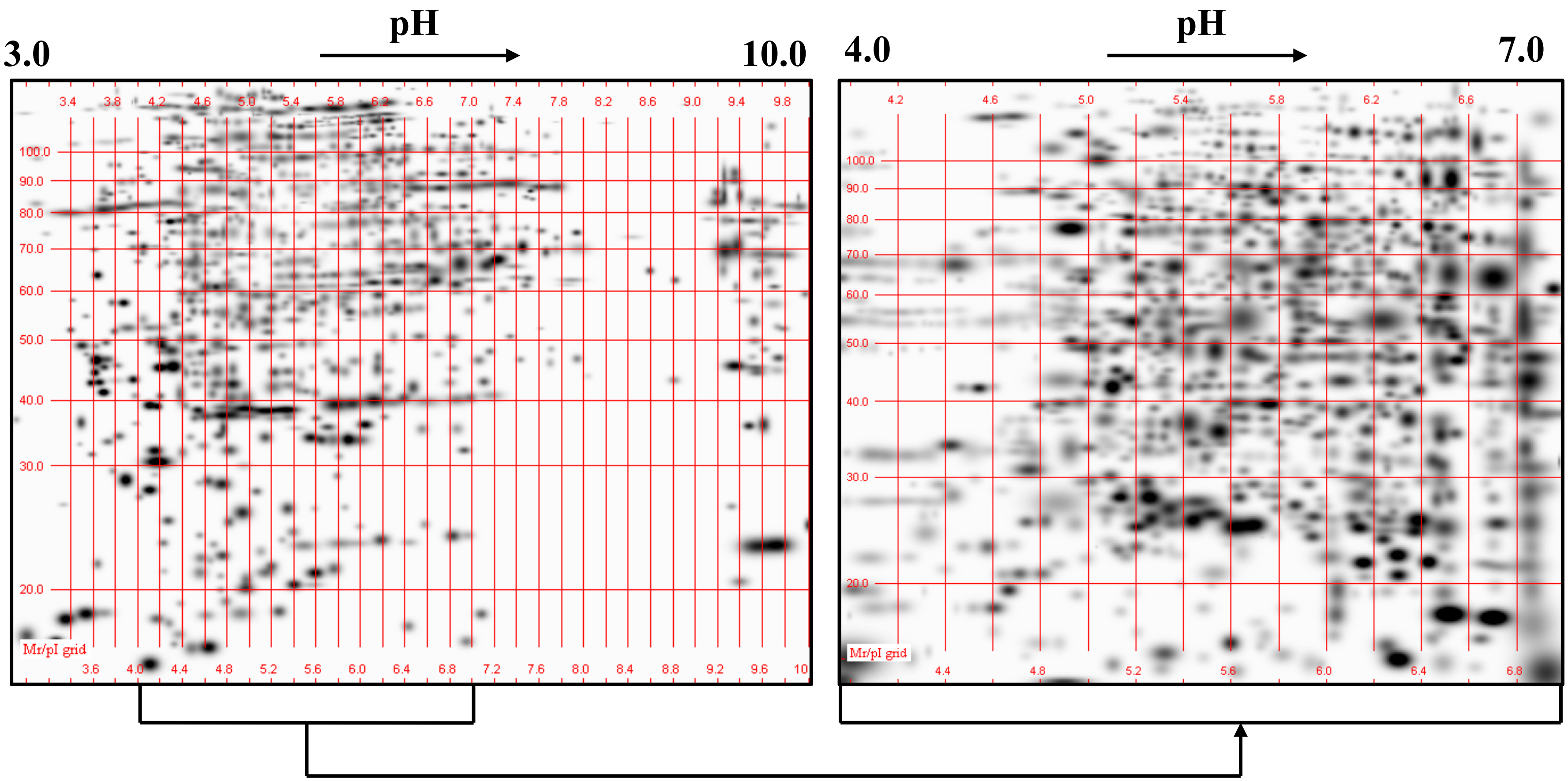

Figure S1. Comparison of protein distribution patterns separated by different precast immobilized pH gradient (IPG) strips. Total proteins isolated from rice seedlings were separated using IPG strips with a pH range of 3-10 (left) and pH 4-7 (right). Note that the majority of proteins were present within the pH range of 4-7.
